# From disease to noninvasive intracranial monitoring

**DOI:** 10.1590/0004-282X-ANP-2021-0298

**Published:** 2022-03-14

**Authors:** Caroline Mensor Folchini, Simone Carreiro Vieira Karuta, Marinei Campos Ricieri, Fábio Araújo Motta, Guilherme de Rosso Manços, Gustavo Frigieri, Adriano Keirijo Maeda

**Affiliations:** 1 Universidade Federal do Paraná, Hospital de Clínicas, Departamento Medicina Interna, Curitiba PR, Brazil.; 2 Instituto de Neurologia de Curitiba, Pesquisa Clínica, Curitiba PR, Brazil.; 3 Hospital Pequeno Príncipe, Núcleo de Pesquisa Clínica, Curitiba PR, Brazil.; 4 Hospital Pequeno Príncipe, Serviço de Neurocirurgia Pediátrica, Curitiba PR, Brazil.; 5 Complexo Pequeno Príncipe, Escritório de Inovação, Curitiba PR, Brazil.; 6 Braincare Desenvolvimento e Inovação Tecnológica, Departamento Científico, São Carlos SP, Brazil.

**Keywords:** Hydrocephalus, Normal Pressure, Parkinson Disease, Monitoring, Hidrocefalia de Pressão Normal, Doença de Parkinson, Monitoramento

## Abstract

Professor Sérgio Mascarenhas was a Brazilian researcher with a vast legacy. His work paved the way for new research possibilities by consolidating the use of innovation and transdisciplinary science. In Medicine, he proposed changes to what had previously been well-accepted concepts, and his contributions have influenced medical practices. Although many authors consider intracranial pressure (ICP) as an unrivaled variable for monitoring and diagnosis of many diseases, its clinical applicability is still the subject of debate in the literature because of the difficulty in standardizing protocols. Mascarenhas's research and the creation of a device for noninvasive monitoring of intracranial compliance are discussed and are shown to have led to the creation of Brain4care, a start-up, and a new perspective on the debate on ICP monitoring.

## RESUMO

O professor Sérgio Mascarenhas foi um pesquisador com vasto legado. Seu trabalho abriu caminho para novas possibilidades de pesquisa, consolidando o uso da inovação e da ciência transdisciplinar. Na Medicina, ele propôs mudanças no que antes eram conceitos bem-aceitos, e suas contribuições influenciaram a prática médica. Embora muitos autores considerem a pressão intracraniana (PIC) uma variável incomparável para o monitoramento e o diagnóstico de uma série de doenças, sua aplicabilidade clínica ainda é motivo de debate na literatura pela dificuldade de padronização de protocolos. A pesquisa do Professor Mascarenhas e a criação de um dispositivo para o monitoramento não invasivo da complacência intracraniana levaram à criação do Brain4care, uma *start-up*, e a uma nova perspectiva sobre o debate do monitoramento da PIC.

## INTRODUCTION

**Figure 1:**
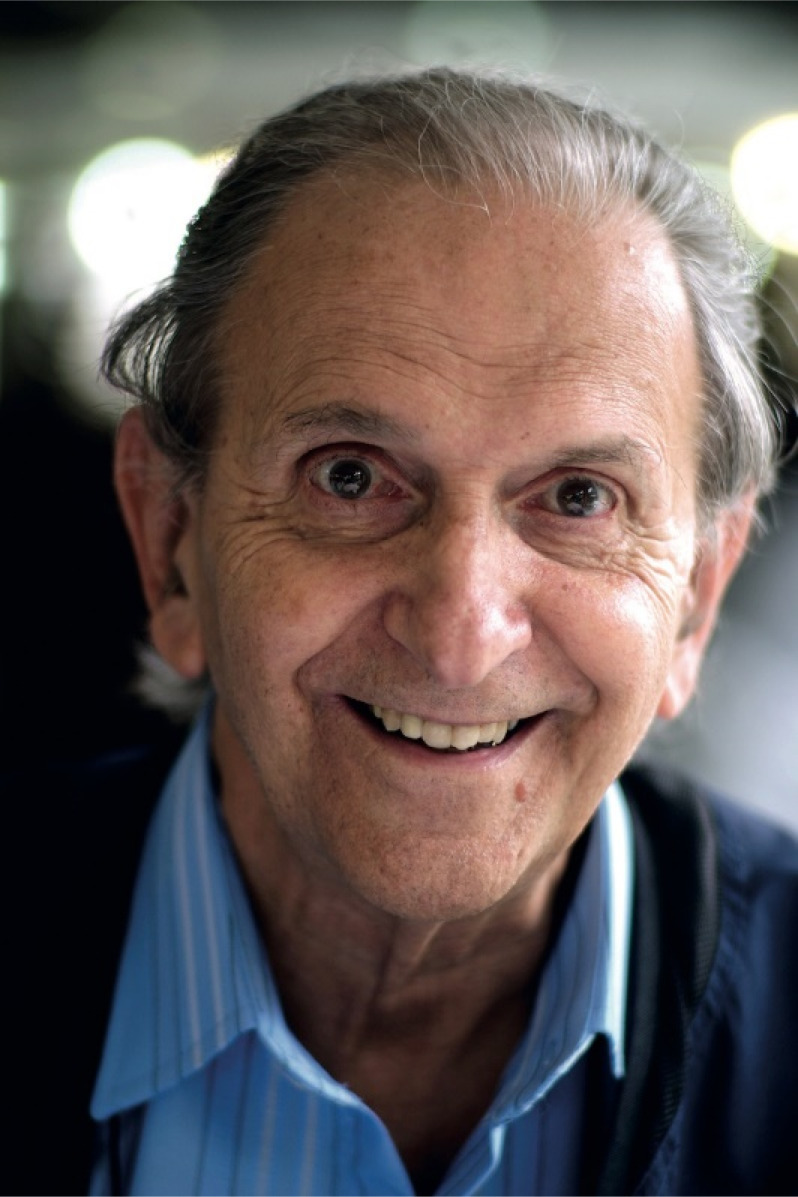
Professor Sergio Mascarenhas.

Professor Sérgio Mascarenhas (May 2, 1928–May 31, 2021), a former professor at the Universidade de São Paulo (USP) and one of the founders of the Universidade Federal de São Carlos (UFSCar) and the Empresa Brasileira de Pesquisa Agropecuária (Embrapa)^1^, was an influential researcher in the field of Health Sciences, and his contribution to this area is set to change the history of Medicine (Figure 1).

In 2006, Mascarenhas, who had previously enjoyed good health, started having symptoms that included severe headache and walking difficulties associated with urinary incontinence. Although an initial diagnostic hypothesis for his clinical condition was parkinsonian dementia, no drug treatment was started, and after a year spent in seeking confirmation of this diagnosis, Mascarenhas finally learned that he had normal pressure hydrocephalus (NPH)^1^.

NPH is a neurological disease that is considered to be reversible and potentially treatable. Although its epidemiology has not yet been established, the incidence is estimated to be 21.9 per 100,000 individuals^2^.

While the classic triad of NPH consists of gait and balance disturbances, changes in urinary control, and cognitive disorders, in the parkinsonian presentation of NPH, cognitive abnormalities have been reported to predominate over motor symptoms in about 30% of patients. One study found that 75% of NPH patients may have overlapping characteristics of dementia, such as those observed in Parkinson's dementia. As a result, the two conditions initially have similar signs and symptoms, giving rise to frequent misdiagnosis, as in the case of Mascarenhas^2–4^.

The treatment chosen for Mascarenhas's condition was a neurosurgical correction of the hydrocephalus with the placement of a ventriculoperitoneal shunt and drainage of excess cerebrospinal fluid (CSF). Having used his experience to investigate the existing technologies for monitoring intracranial pressure (ICP), Mascarenhas was surprised to find that the only methods that had been validated were invasive ones. Unwilling to accept this, Mascarenhas started a study that led not only to the development of a device that would influence medical practice but also to the changes in what had previously been well-established concepts.

From his diagnosis to the development of the device, this Brazilian scientist traveled a long, arduous, yet undoubtedly brilliant path, and today the fruits of his phenomenal work are being reaped.

## MASCARENHAS AND NORMAL PRESSURE HYDROCEPHALUS

The Monro-Kellie doctrine (1783) states that the sum of the volumes of CSF, blood, and brain parenchyma (intracranial) must be constant and that an increase in one of these components should generate a reduction in one or more of the others for intracranial abnormalities not to occur and volume equilibrium to be maintained. This doctrine also implies that once a child's fontanelles have closed, the cranial volume remains constant, i.e., there is no cranial deformation secondary to changes in intracranial components after this period^5,6^.

Mascarenhas and his fellow researchers proved in 2007 that cranial deformities caused by increased internal pressure in the brain can be detected, raising several questions about the implications of the Monroe–Kellie doctrine.

Mascarenhas and his colleagues initially performed tests *in vitro*. Strain sensors used in civil engineering (strain gauges) were glued to a human skullcap. The skull was filled with a rubber balloon connected to a bulb pump device, and the balloon was inflated to validate the method statistically^4,5^.

The second phase included *in vivo* experiments, which were performed with changes in the degree of elevation of the animals’ heads (30°, 45°, and 90°). Corroborating the findings in the literature, the experiment demonstrated that postural changes lead to variations in ICP and that the skull is indeed expandable, even after the closure of the fontanelles. The experiments also showed that this deformation can be detected with a sensor placed on the scalp without the need to insert a catheter to monitor ICP^4,5^.

As there is a dearth of class A studies on ICP monitoring in clinical research, the use of this technique is still the subject of debate. Furthermore, as existing studies use different methodologies to measure ICP, their results cannot be meaningfully compared.

There are several invasive and noninvasive techniques for measuring ICP in Table 1. However, the current gold standard involves the placement of an intraventricular drain that is connected to an external fluid pressure sensor, which also provides therapeutic drainage of CSF. Another invasive method involves the insertion of parenchymal or intraventricular sensors. Both techniques expose the patient to risks. These and other methods, as well as the advantages and limitations of each, are summarized in Table 1. The CSF tap test, a less invasive method, is also used widely in the clinical environment to determine ICP (cmH_2_O)^7–10^.

**Table 1:** Comparison of intracranial pressure monitoring techniques.

Technique	Classification	Strengths and limitations
Ventricular catheter	Invasive	Measures global ICP. 2. Can be recalibrated. 3. Can drain cerebral fluid. 4. Increased risk of infection.
Intraparenchymal catheter	Invasive	1. Higher cost. 2. Can be calibrated. 3. Cerebral fluid cannot be drained. 4. Risk of infection.
Implantable ICP sensors	Invasive	Risk of bleeding and infection.
Telemetry sensors and miniature sensors	Invasive	1. Biocompatible electronics, biocompatible energy sources, and efficient telemetry. 2. Bleeding and risk of infection.
Biodegradable pressure sensors	Invasive	1. Risk of infection.
Lumbar puncture	Invasive	1. If CSF pathways are occluded, ICP will not be measured correctly. 2. Risk of infection.
Monitoring of epidural ICP	Invasive	Risk of bleeding is reduced. 2. Risk of infection.
Transcranial Doppler	Noninvasive	1. Highly user-dependent. 2. Small changes in probe direction can significantly affect the Doppler signal. 3. Affected by other changes in physiology such as medication-related changes, autoregulation, and hyperemia.
Optical methods	Noninvasive	Depends on a patent cochlear aqueduct, which serves as a mechanical filter for transmitting ICP-derived signals.
Image-based methods	Noninvasive	1. Provides only short term assessments. 2. Repetitive measurements not possible. 3. Expensive and not available in many settings.
Acoustic methods	Noninvasive	1. There are no validated acoustic methods for measuring ICP noninvasively.
Optic nerve sheath diameter	Noninvasive	Manipulator-dependent. 2. Ability to separate high and low ICP makes it primarily a screening tool and less valuable at the bedside.
Brain4care	Noninvasive	Manipulator-dependent. 2. Monitoring in presurgical patients.

ICP: intracranial pressure; CSF: cerebrospinal fluid.

In terms of noninvasive monitoring, several techniques have been developed, such as the use of transcranial Doppler, the measurement of the diameter of the optic nerve, and the use of biodegradable sensors. However, these methods all measure ICP indirectly^7–9^.

The noninvasive device developed by Mascarenhas for real-time ICP monitoring has been validated technically, and comparisons between invasive methods and the new device have been undertaken. When the signal from the sensor used in the noninvasive device developed by Mascarenhas is plotted, the resulting curve is very similar to that obtained using invasive ICP monitoring. The pulse pressure curve is subdivided into three waves: P1 — the percussion wave, which is related to the arterial pulse transmitted to the choroid plexus and is usually the largest peak; P2 — the tidal wave, which is related to the compliance of brain tissue; and P3 — the dicrotic wave. Final compliance is given by the ratio p2/p1^6,7^.

In the last six years, Mascarenhas's research group has published papers showing the effectiveness of their sensor in clinical practice and adult patients. At the same time, research has been undertaken into the pediatric use of the device^10–19^.

Mascarenhas's research has contributed a new perspective to the debate on ICP monitoring. The device developed by Brain4care, a start-up, as a result of Mascarenhas’ research is a new tool for noninvasive monitoring of intracranial compliance that improves the quality of life of patients without exposing them to possible complications from invasive procedures.

The decision to use his own medical condition to develop a device that can help in both the diagnosis and treatment of patients with conditions that are often disabling is undoubtedly Mascarenhas's great legacy in the field of medical science.
